# Evaluation of Metaxylem Vessel Histogenesis and the Occurrence of Vessel Collapse during Early Development in Primary Roots of *Zea mays* ssp. *mexicana*: A Result of Premature Programmed Cell Death?

**DOI:** 10.3390/plants9030374

**Published:** 2020-03-18

**Authors:** Susumu Saito, Teruo Niki, Daniel K. Gladish

**Affiliations:** 1 Department of Biotechnology, Takushoku University, Tatemachi 815-1, Hachioji, Tokyo 193-0985, Japan; mdjmk210@ybb.ne.jp (S.S.); tniki@la.takushoku-u.ac.jp (T.N.); 2 Department of Biology, Miami University, 1601 University Blvd, Hamilton, OH 45011, USA

**Keywords:** metaxylem, root development, vessel collapse, programmed cell death, *Zea mays*

## Abstract

Root apical meristem histological organization in *Zea mays* has been carefully studied previously. Classical histology describes its system as having a “closed organization” and a development of xylem that conforms to predictable rules. Among the first cell types to begin differentiation are late-maturing metaxylem (LMX) vessels. As part of a larger study comparing domestic maize root development to a wild subspecies of *Z. mays* (teosinte), we encountered a metaxylem development abnormality in a small percentage of our specimens that begged further study, as it interrupted normal maturation of LMX. Primary root tips of young seedlings of *Zea mays* ssp. *mexicana* were fixed, embedded in appropriate resins, and sectioned for light and transmission electron microscopy. Longitudinal and serial transverse sections were analyzed using computer imaging to determine the position and timing of key xylem developmental events. We observed a severe abnormality of LMX development among 3.5% of the 227 *mexicana* seedlings we screened. All LMX vessel elements in these abnormal roots collapsed and probably became non-functional shortly after differentiation began. Cytoplasm and nucleoplasm in the abnormal LMX elements became condensed and subdivided into irregularly-shaped “macrovesicles” as their cell walls collapsed inward. We propose that these seedlings possibly suffered from a mutation that affected the timing of the programmed cell death (PCD) that is required to produce functional xylem vessels, such that autolysis of the cytoplasm was prematurely executed, i.e., prior to the development and lignification of secondary walls.

## 1. Introduction

The cellular organization of the apical meristems of primary roots of flowering plants has been described as being “closed” or “open” [1,2,3] depending upon whether mitotically active initials (stem cells) are associated at the margins of distinct layers of cells in the promeristem, called histogens [3,4], or not.

Plants in the Poaceae (grasses) family typically have root apical meristems with a closed organization, and have been most often described as having three defined histogen layers. In this scheme for grass root apices, the root cap-producing calyptrogen is usually quite distinct and the protoderm and ground meristem are produced by a single dermatogen/periblem complex. The pericycle and procambial tissues of the stele are produced by the plerome [3,5]. On the other hand, in a recent study on *Zea mays,* we reported that in the two subspecies that were examined, *Z. mays* “Honey Bantam” (a sweetcorn cultivar) and *Z. mays* ssp. *mexicana* (a wild teosinte), the plerome only produced the pericycle, and a fourth biseriate layer basipetal to the plerome produced the rest of the procambium by the proliferation of cells basipetally. We described this as the “vascular initials zone” and suggested the name “vasculogen” for this apparent fourth histogen [6]. We also reported that the first two vascular cell types to become distinguishable by the development of *Z. mays* stele were pericycle cells and vessel elements of late-maturing metaxylem (LMX). LMX vessels could often be traced to initials within the vascular initials zone [6].

In the process of examining *Z. mays mexicana*, we encountered a severe abnormality in the development of the LMX vessels of the primary roots in a small percentage of the seedlings. Root vessels in the abnormal seedlings collapsed early in development, and given that vessel element differentiation concludes with programmed cell death (PCD) [7], we hypothesized that the vessel collapse in abnormal *Z. mays* ssp. *mexicana* roots was due to mistimed PCD. The present paper reports the results of our morphological study of this abnormality by comparison to normal vascular development in *Z. mays mexicana*, and presents a discussion of its implications for understanding the molecular control of xylem development and potential seedling pathogenesis in maize.

## 2. Results

### 2.1. Normal Meristem and Xylem Development

**Figure 1:**
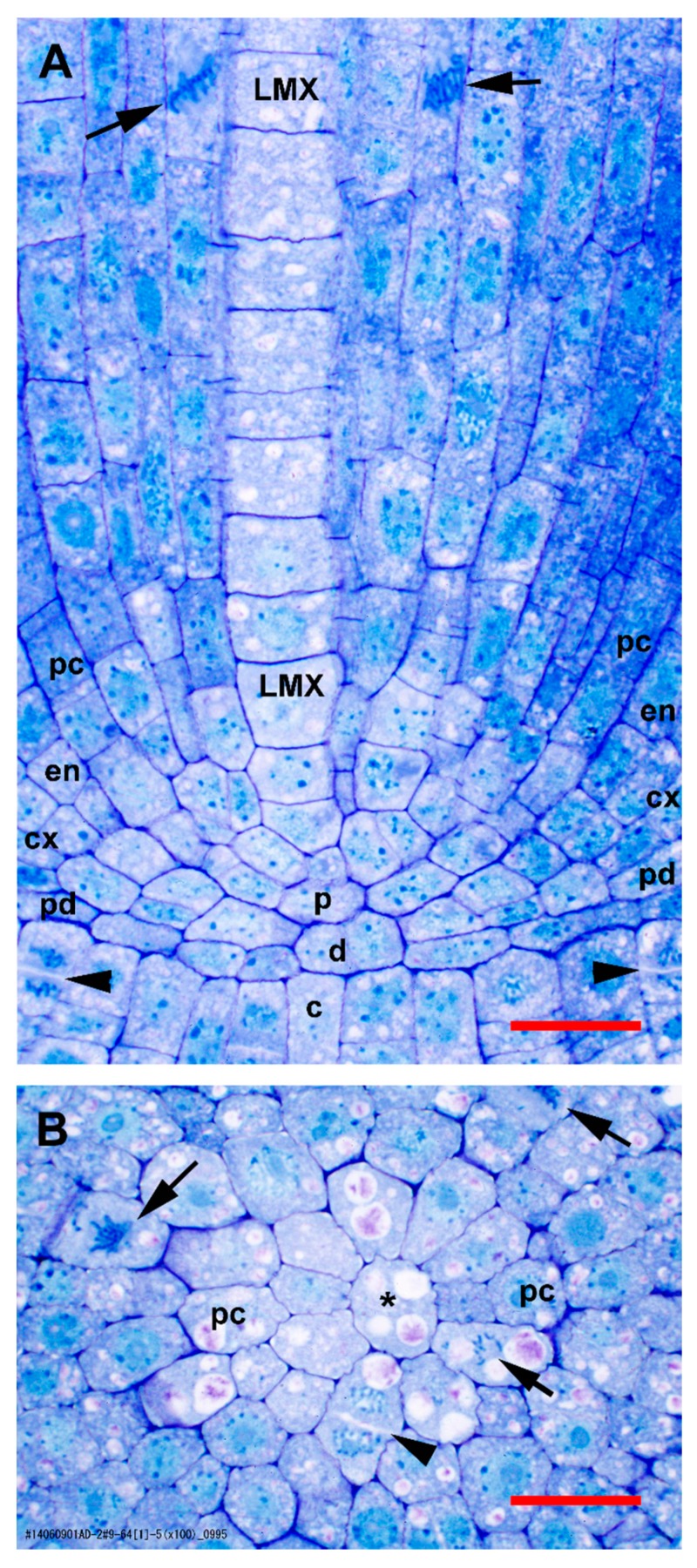
Normal organization of tissues in the promeristem and primary meristem zones of teosinte roots at high magnification. (**A**) Slightly off-median longitudinal section. (**B**) Transverse section through the plerome showing a margin initial during cytokinesis (arrowhead) and another in mitosis (arrow). Late-maturing metaxylem (LMX) vessel; asterisk (*), plerome central cell; arrow, mitotic cell; arrowhead, newly forming cell plate; c. calyptrogen; d. dermatogen/periblem complex; p, plerome; cx, cortex; en, endodermis; pc, pericycle; pd, protoderm; rc, rootcap. Scale bar = 25 µm. Modified from Saito et al. 2019 [6], cf. Figure 1.

The root apical meristem of *mexicana* teosinte has the closed anatomical organization typical of most grasses and is similar to that of domestic maize (Figure 1).

Based on staining density differences and a general lack of mitotic figures and new cell walls, the quiescent center (QC) was mainly limited to and occupied by the 7–12 cells of the dermatogen/periblem complex and the 7–9 cells of the plerome that were centrally located at the end of the root body proper (Figure 1). These histogens were usually organized as a distinct central cell surrounded by a ring of additional cells which we called margin initials, all of which had lower stain densities than cells farther away from the center (Figure 1). Sometimes, the plerome cells contained vacuoles with inclusions and other times not, which suggests cyclic behavior (compare Figure 1A to Figure 1B). Ground meristem and protoderm could be distinguished within one cell of a dermatogen/periblem margin initial by virtue of a periclinal division of its first derivative. Subsequent periclinal divisions of the inner of those derivative cells initiated the differentiation of the endodermis from the rest of the ground meristem of the cortex (Figure 1A).

**Figure 2:**
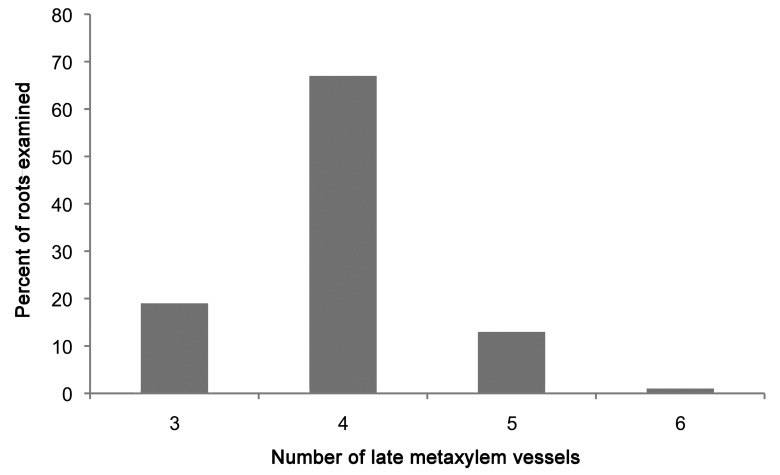
Distribution of numbers of late metaxylem vessels per primary root among 227 teosinte seedlings.

**Figure 3:**
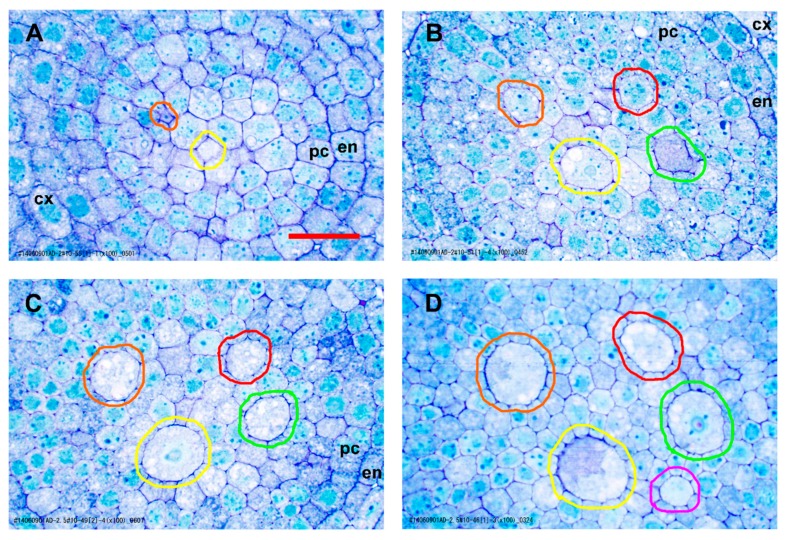
Normal LMX and first early metaxylem (EMX) development revealed by representative transverse sections from among ca. 600 serial sections taken from one teosinte primary root. The micrographs are centered on the stele. (**A**) Appearance of first (encircled yellow) and second (orange) LMX (38 µm from the rootcap junction [RCJ]). (**B**) All four LMX present (78 µm from RCJ); third (green), fourth (red). (**C**) 110 µm from RCJ. (**D**) First EMX (magenta) appeared by 204 µm from RCJ. EMX, early metaxylem; LMX, late metaxylem vessel; cx, cortex; en, endodermis; pc, pericycle. Scale bar = 25 µm.

In the procambium, the pericycle cells could be differentiated earliest, and these proliferated directly from the margin initials of the plerome mainly by tangential divisions anticlinal to the plane of the histogen (Figure 1B). The remainder of the procambium was produced by cell divisions in the two, somewhat irregularly organized, layers of initial cells immediately basipetal to the plerome (Figure 1A). So named because they are the last to mature and become functional [3,5], the late-maturing metaxylem (LMX) vessel elements were the second vascular cell type to become recognizable. Most primary roots developed four large LMX vessels (Figure 2 and Figure 3), beginning within a few cells of the plerome (ca. 35 µm from the rootcap junction [RCJ]), and later differentiating early metaxylem (EMX) vessels 75–200 µm from the RCJ (Figure 3D). The first of the LMX elements began differentiating immediately upon being produced by the more basipetal of the two vascular initial layers, i.e., within one or two cells of the plerome (Figure 1A). LMX always began differentiation in a staggered fashion. The second LMX vessel typically became detectable within only 10 µm farther from the initials that began the first (Figure 3A). Viewed in transverse section, the later-differentiating LMX element became detectable in no particular orientation with respect to the first. Once LMX differentiation had begun, axially-oriented cell divisions in various planes occurred among the parenchymatous cells in the central area of the stele until each LMX vessel was usually surrounded by a distinct ring of cells of roughly the same size and shape (Figure 3B–D).

### 2.2. Abnormal Xylem Development

**Figure 4:**
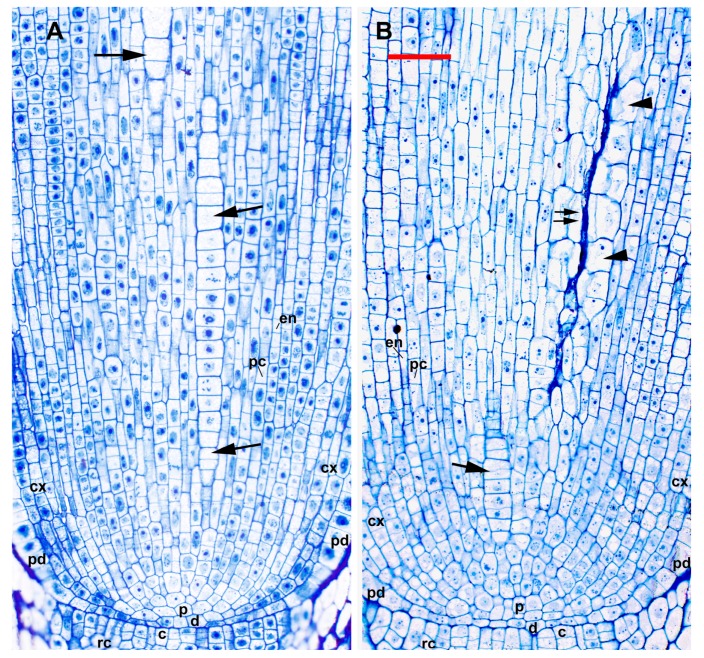
Off-median longitudinal sections showing (**A**) normal LMX (arrows) and (**B**) abnormal (double arrows) LMX vessel development in teosinte root tips. During early development abnormal LMX appear normal. Xylem parenchyma cells surrounding the collapsed elements of an abnormal vessel (arrowheads) are distinctly enlarged and irregular. c, calyptrogen; d. dermatogen/periblem complex; p, plerome; cx, cortex; pc, pericycle; pd, protoderm, en, endodermis; rc, rootcap. Scale bar = 50 µm.

**Figure 5:**
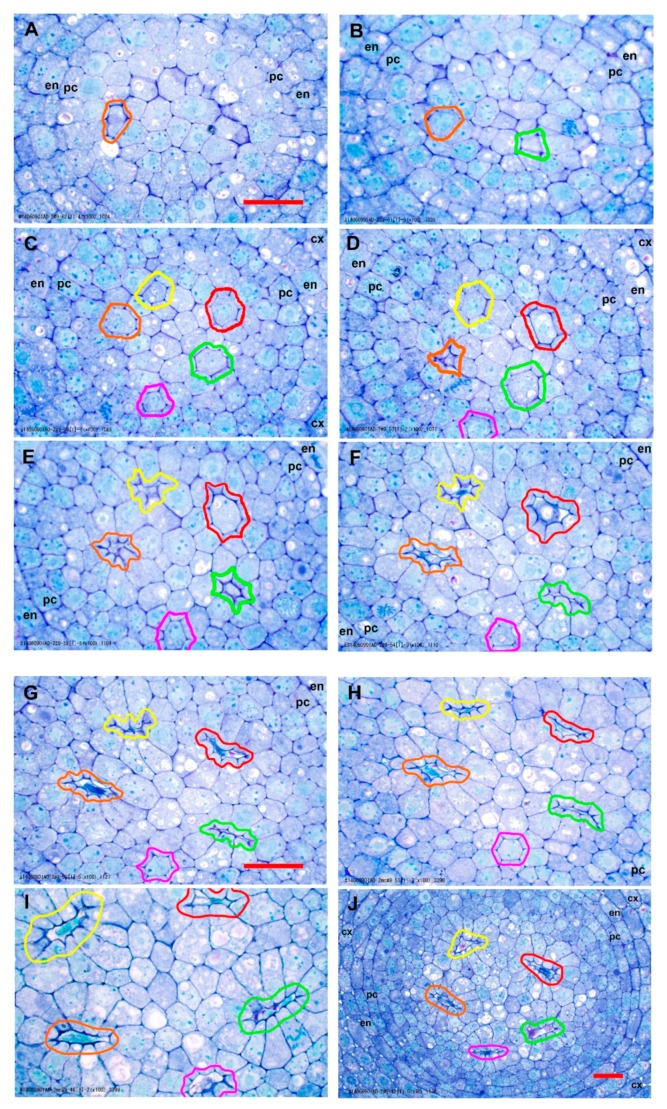
Abnormal LMX and first EMX development revealed by representative transverse sections from among ca. 335 serial sections taken from one teosinte primary root. (**A**) Appearance of first LMX element (orange) 34 µm from RCJ. (**B**) Appearance of the second LMX element (green) 46 µm from RCJ. (**C**) The third (red) and fourth (yellow) LMX elements were differentiated by 78 µm from RCJ, and all elements appeared normal. (**D**) The first-formed LMX vessel (orange) began collapsing by 92 µm from the RCJ, about the same time the first EMX element (magenta) could be discerned. A cell adjacent to the collapsing element was in mitosis. (**E**) The second (green) and fourth (yellow) LMX elements began collapsing by 106 µm from RCJ. Cells adjacent to collapsing vessels were obviously enlarged and became radially lengthened in shape. (**F**) The third LMX vessel (red) was collapsing by 120 µm from RCJ. Its cytosol appeared plasmolyzed. (**G**) All four LMX were nearly or completely collapsed by 126 µm from RCJ, and condensed cytosol was seen in some of them. (**H**) All four LMX were collapsed, but the EMX element appeared normal 132 µm from the RCJ. (**I**) At 202 µm from RCJ, it was seen that radial growth of the parenchymatous cells had continued as normal, but all the cells adjacent to collapsing vessels were abnormally enlarged by lengthening into the space yielded by the collapsing vessels. The EMX vessel had begun to collapse and appeared to be plasmolyzed. (**J**) Lower magnification view of the vascular tissue at 236 µm. Phloem differentiation and further EMX differentiation were not yet apparent. cx, cortex; en, endodermis; pc, pericycle. Scale bar = 25 µm.

**Figure 6:**
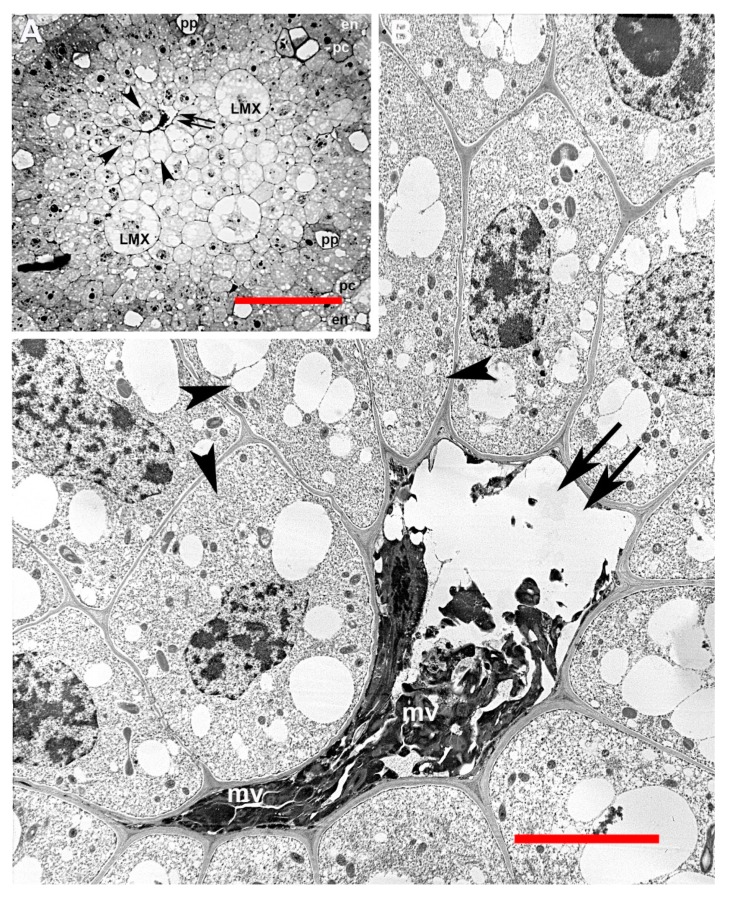
Collapse of LMX during abnormal development. (**A**) Light micrograph of a transverse section with one of four LMX elements in a state of collapse. Scale bar = 50 µm. (**B**) Highly magnified transmission electron micrograph featuring the same abnormal vessel element as shown in Figure 6A filled with dense, irregular macrovesicles (mv). LMX, unaffected late metaxylem vessel element; double arrow, collapsing late metaxylem vessel element; arrowhead, distorted and enlarged xylem parenchyma cells surrounding a collapsing late metaxylem vessel element; pc, pericycle; en, endodermis; pp, protophloem. Scale bar = 10 µm.

**Figure 7:**
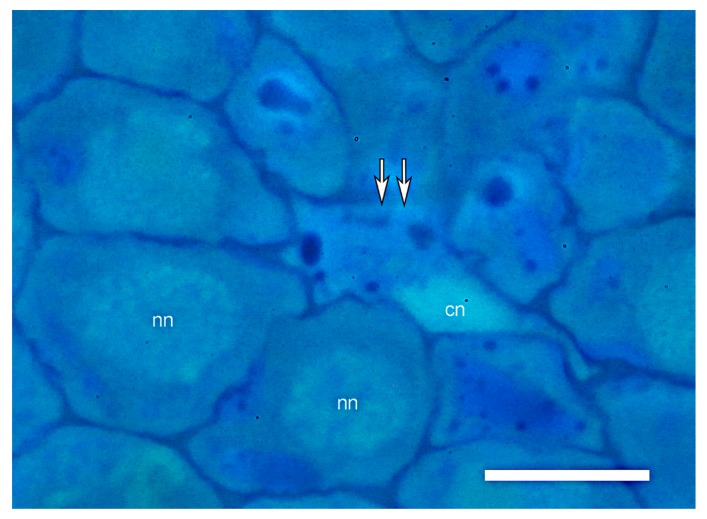
Condensed chromatin in the nucleus of a collapsed LMX element. Highly magnified, DAPI-stained transverse section viewed via fluorescent light microscopy reveals that chromatin in nuclei of a collapsing LMX element (double arrows) is highly condensed compared to nuclei of surrounding cells. nn, normal nucleus; cn, condensed nucleus. Scale bar = 10 µm.

Nine of the 227 primary roots that were microscopically examined (3.5%) showed evidence of premature death and collapse of LMX and EMX. Roots that had abnormal metaxylem development had normal promeristem organization with a typical QC. The progression of cell proliferation and the establishment of the primary meristem tissues appeared completely normal (Figure 4). No cell types were visibly affected except LMX and EMX elements and the parenchymatous cells immediately surrounding them, though roots with this abnormality began differentiation of phloem sieve tubes about 200 µm farther from the RCJ than normal roots. LMX elements proliferated from initials in the vascular initials zone (vasculogen) basipetal to the plerome in a distinct file. These elements then grew and subsequently divided transversely one to three times in a normal fashion, usually until they were 45–100 µm from the first differentiating cell in their cell file, when they began to collapse (Figure 4B and Figure 5D–J). Collapsing cells often appeared plasmolyzed and appeared to have condensed, disorganized nuclei, degraded organelles, a thinned primary cell wall, and a cytoplasm fragmented into irregular “macrovesicles” (Figure 5, Figure 6 and Figure 7). These vessel elements apparently lost turgor, as cells immediately surrounding them enlarged centripetally into space previously occupied by the vessel, which caused the surrounding cells to take on an irregular form (Figure 4).

## 3. Discussion

It has been reported that as tracheary elements complete their differentiation, the cytoplasm breaks up into “cytoplasmatic spherules”, some of which contain organelles, that enter the central vacuole and ultimately become digested [7,8,9]. In the present study, the cytoplasm in the LMX elements of the abnormally developing steles was observed to become condensed and subdivided into irregularly-shaped macrovesicles seemingly pressed together as the cells collapsed inward (Figure 6B). We speculate that these macrovesicles are analogous to the cytoplasmatic spherules reported by Wodzicki and Humphries [8,9], but are malformed presumably as a consequence of autolysis being executed prematurely.

Our serendipitous discovery of a significant developmental abnormality limited strictly to metaxylem vessel elements will provide, we think, an unusual opportunity to explore a key aspect of the molecular regulation of metaxylem vessel differentiation, since it is so specific in cell type and timing. The small number of affected individuals (3.5%) among our population of seedlings suggests that the cause might be a recessive single gene mutation carried by a small number of plants in the field population from which the seeds were collected. That it has a synchronized timing among the affected metaxylem vessels suggests that it might be a “control” gene. It is known that programmed cell death (PCD) is involved in the maturation of xylem tracheary elements in plants [7,10]. However, the PCD process in LMX, which takes a long time to complete growth to a large size and then develop thick secondary walls and autolyse [3,5], is not usually detected at such an early stage of development as that of the cells we observed collapsing. The collapsing LMX had morphologically distorted and condensed nuclei (Figure 7) [10], but showed no evidence of incipient secondary wall deposition. In fact, the primary walls had thinned (Figure 6), which is a characteristic of some plant PCD [11,12]. So, perhaps this is a case of a precocious induction of PCD leading to the loss of membrane integrity, and therefore turgor pressure, before a secondary wall has begun to form that could resist the pressure resulting from the continued turgor within the surrounding growing parenchymatous cells. Parenchyma cells in xylem tissue are known to press or grow into vascular cavities formed by PCD in roots of legumes [13] and into the lumena of tracheary elements through pits in older, non-functioning secondary xylem vessels during tylosis formation [3]. In either case, the phenomenon we report here may be due to a genetic misregulation of normal developmental functions. A mutation that alters, among other things, the normal tissue distribution that results in amphivasal vascular bundles has been reported in *Arabidopsis thaliana* [14]. A mutation affecting xylem development that interferes with water transport has also been identified in *Arabidopsis* [15], but the phenotype is not as anatomically severe as the one we report here. We have not encountered published reports describing anything like our observations in *Zea mays mexicana*.

## 4. Conclusions

The abnormality was observed among seedlings grown from commercial seed obtained from plants grown in the open environment. Since it was found in seedlings grown from randomly selected seed from a commercial source under non-stressful conditions, the abnormality appears to be heritable. *Z. mays mexicana* is known to freely hybridize with conventional *Z. mays* [16,17], and *Z. mays* is a wind-pollinated species. Further work is needed to determine if the abnormality is severe enough to prevent reproduction or is ultimately fatal, but if that is the case there is a possibility it could, or already has, spread by hybridization into conventional maize populations and may contribute to reduced viability of some seedlings in cultivation. Admittedly, such a project would be a challenge, given that no specific information on the parental lines was available for us to share with readers. On the other hand, the vendor and seed lot number are known, as is the nation where the seeds were produced, so it is possible that seeds similar to ours can be obtained for further study of this phenomenon. This species produces abundant seminal adventitious roots from the mesocotyl. Therefore, sampling, while it is destructive to the primary root, can be done without killing the germinating seedling. Although, maintaining such plants might have to be done via laboratory culture because of the severity of the vascular abnormality.

## 5. Materials and Methods

### 5.1. Plant Material

Cultivation methods were modified after Gladish and Niki [18]. Teosinte seeds (*Zea mays* L. ssp. *mexicana* (Poaceae), lot #K0018 raised in Thailand, Snow Brand Seed Co. Ltd, Sapporo, Japan) were surface sterilized in 10% (v/v) household bleach. Seeds were sown in 1 L autoclaved beakers filled with autoclaved, moistened vermiculite (375 mL water/L vermiculite) and covered with sterilized foil under axenic conditions. The beakers were placed in a continuously dark growth chamber at a constant temperature of 25 °C. After 3 d, primary roots were collected.

### 5.2. Light Microscopy (LM)

Procedures were modified from Niki et al. [19]. For conventionally prepared specimens, 3–4 mm long root tip segments were immediately immersed in 4% (w/v) paraformaldehyde in 0.1 M phosphate buffer and gently shaken overnight at room temperature. Following fixation, the segments were rinsed in buffer, dehydrated by ethanol series, embedded in Technovit 7100™ resin (Heraeus Kulzer GmbH, Wehrheim, Germany) and sectioned at 2 or 2.5 µm (transverse and longitudinal, respectively) for LM on a Leica Ultracut UCT ultramicrotome (Leica Ltd, Tokyo, Japan). These were mounted on slides and stained with 0.1% (w/v) toluidine blue O or with 0.1% DAPI (4’, 6-diamidino-2-phenylindole dihydrochloride). Sections were viewed with a Leica DMLB light microscope (Leica Microsystems, Wetzlar, Germany) equipped with Nikon TU Plan Fluor lenses (Nikon) and photographed with a Canon EOS 5D digital camera (Canon Ltd, Tokyo, Japan).

Roots of 59 seedlings were embedded and sampled by preliminary partial sectioning. From these, five normal and nine abnormal primary roots were selected for detailed study. Using the strategy pioneered by Heimsch [20], 320 to 600 2 µm thick transverse serial sections of the entire tip of each of these roots were painstakingly retained in order when mounted. The center of each root section was photographed at high and very high magnification (125x and 250x, respectively). The resulting digital micrographs were rotated by computer so they had a common alignment. Then, beginning with the most basipetal section, vessel element lineages were carefully traced toward the tip until they could no longer be discerned.

### 5.3. Transmission Electron Microscopy (EM)

Procedures for EM were per Niki et al. [19]. Root tip segments (2–3 mm long) of abnormally-developing roots were immediately immersed in 2.4% (v/v) glutaraldehyde/0.3% (w/v) paraformaldehyde in 0.02 M phosphate buffer (pH 7.2) and gently shaken overnight at room temperature, then immersed in 0.025% (w/v) tannic acid for 3 h. Specimens were post-fixed in 0.1% (w/v) osmium tetroxide at 4 °C for 1 h. After fixation the segments were rinsed several times in buffer before being rinsed in distilled water, dehydrated by alcohol series, and embedded in Spurr epoxy resin. Embedded specimens were sectioned transversely at 0.07 µm, stained with saturated uranyl acetate and Reynold’s lead citrate solution, and mounted on copper specimen grids for observation using a Hitachi H300S transmission electron microscope (Hitachi Ltd., Tokyo, Japan) at 75 KV. Some 2 µm sections were made afterward from the same block, stained with toluidine blue O, and used for comparative LM (Figure 7A).

### 5.4. Graphics 

Figures were prepared using Photoshop™ CS5 and saved in TIFF format.
